# Voluntary self-touch increases body ownership

**DOI:** 10.3389/fpsyg.2015.01509

**Published:** 2015-10-27

**Authors:** Masayuki Hara, Polona Pozeg, Giulio Rognini, Takahiro Higuchi, Kazunobu Fukuhara, Akio Yamamoto, Toshiro Higuchi, Olaf Blanke, Roy Salomon

**Affiliations:** ^1^Graduate School of Science and Engineering, Saitama UniversitySaitama, Japan; ^2^Center for Neuroprosthetics, École Polytechnique Fédérale de LausanneLausanne, Switzerland; ^3^Neuroscience, Brain Mind Institute, École Polytechnique Fédérale de LausanneLausanne, Switzerland; ^4^School of Engineering, École Polytechnique Fédérale de LausanneLausanne, Switzerland; ^5^Department of Health Promotion Sciences, Tokyo Metropolitan UniversityTokyo, Japan; ^6^Department of Precision Engineering, School of Engineering, The University of TokyoTokyo, Japan; ^7^Department of Neurology, University Hospital of GenevaGeneva, Switzerland

**Keywords:** sense of body ownership, sense of agency, self-touch, rubber hand illusion, multisensory integration, volition, robotics and haptic technology

## Abstract

Experimental manipulations of body ownership have indicated that multisensory integration is central to forming bodily self-representation. Voluntary self-touch is a unique multisensory situation involving corresponding motor, tactile and proprioceptive signals. Yet, even though self-touch is frequent in everyday life, its contribution to the formation of body ownership is not well understood. Here we investigated the role of voluntary self-touch in body ownership using a novel adaptation of the rubber hand illusion (RHI), in which a robotic system and virtual reality allowed participants self-touch of real and virtual hands. In the first experiment, active and passive self-touch were applied in the absence of visual feedback. In the second experiment, we tested the role of visual feedback in this bodily illusion. Finally, in the third experiment, we compared active and passive self-touch to the classical RHI in which the touch is administered by the experimenter. We hypothesized that active self-touch would increase ownership over the virtual hand through the addition of motor signals strengthening the bodily illusion. The results indicated that active self-touch elicited stronger illusory ownership compared to passive self-touch and sensory only stimulation, and show an important role for active self-touch in the formation of bodily self.

## Introduction

A fundamental aspect of the experience of the self is the sensation that we have a body (body ownership) and that we control the actions of that body (agency) (Merleau-Ponty, 1996; Gallagher, 2000; Jeannerod, 2003). Body ownership is based on the correspondence and integration of multisensory signals (e.g., Ehrsson et al., 2004; Blanke, 2012; Salomon et al., 2013b). Agency, the sense of control over one's own actions, is thought to rely on integration of efferent and afferent sensorimotor information (Blakemore and Frith, 2003; Haggard, 2005; David et al., 2008; Jeannerod, 2009). A particular and relevant case in which body ownership and self-generated action interact is self-touch. Self-touch is thought to engender a basic form of self-awareness (Gallagher and Meltzoff, 1996; Merleau-Ponty, 1996), and has been suggested to contribute to structural and conscious representations of the body (Dieguez et al., 2009; Schütz-Bosbach et al., 2009; Kammers et al., 2010; van Stralen et al., 2011; Blanke et al., 2014). Self-touch is an important cue for body ownership since it includes a multisensory correspondence between two simultaneous tactile inputs (e.g., the hand that is touching and the hand that is being touched) coupled with corresponding motor and proprioceptive signals. Self-touch thus uniquely specifies one's own body as distinct from other objects in the environment. Developmentally, discrimination of self-touch vs. external touch arises early in life. Indeed, it has been shown that infants can discriminate self-touch from external touch when they are only 24 h old (Rochat and Hespos, 1997) and self-touch has been suggested to be important for the development of the sense of self in infancy (Rochat, 1998). Investigation of self-touch revealed that self-generated action reduces the perceived intensity of the tactile stimulation occurring simultaneously with the action (Weiskrantz et al., 1971; Blakemore et al., 1998, 1999). These findings speak in favor of a predictive “forward model” in which the expected sensory consequences of self-generated action are attenuated in order to enhance perception of external events (Wolpert et al., 1995; Bays et al., 2006; Shergill et al., 2012). Other research has linked passive self-touch and body representation through an adapted version of the RHI paradigm (“Somatic RHI,” Ehrsson et al., 2005). In the Somatic RHI, the experimenter uses one of the blindfolded subject's fingers to touch a fake hand while synchronously touching the participant's other hand. This causes the sensation that the subject is touching his own hand and is associated with the mislocalization of the subject's touched hand toward the position of the fake hand (Ehrsson et al., 2005; White et al., 2010; Davies et al., 2013; Pozeg et al., 2014). Previous studies have focused on passive self-touch, yet in real life self-touch is typically caused by voluntary movements and thus includes predictive efferent signals, which may have an important role in establishing bodily self-representation through sensorimotor correspondences. Two contrary predictions could be made regarding the effects of active self-touch on the RHI: First, as active self-touch is associated with an attenuation of subjective tactile intensity and neural activation though efferent signals (e.g., Bays et al., 2005; Shergill et al., 2012) one could expect a reduced illusion due to attenuation of the tactile signal. Contrarily, efferent signals may boost the illusion though the addition of sensorimotor correspondences binding the tactile feedback to the self.

Here, in three experiments, we tested the effects of active and passive self-touch on body ownership. We hypothesized that active self-touch would induce stronger illusory self-ownership in the somatic and visual versions of the RHI due to the addition of efferent signals providing additional sensorimotor correspondences. We employed both subjective measures of illusory self-touch and illusory self-ownership (Botvinick and Cohen, 1998; Ehrsson et al., 2005; Tsakiris and Haggard, 2005; Lenggenhager et al., 2007; Rohde et al., 2011; Kalckert and Ehrsson, 2012; Pozeg et al., 2014) as measured by questionnaires and objective measures of proprioceptive drift (Botvinick and Cohen, 1998; Tsakiris and Haggard, 2005; Costantini and Haggard, 2007; Lenggenhager et al., 2007; Kammers et al., 2009; Tsakiris, 2010) which are well-established measures of bodily illusions (but see Rohde et al., 2011). Across three experiments and two variants of the RHI we found that active self-touch enhanced the illusory ownership.

## Materials and methods

### Participants

Forty participants were recruited through an advertisement at The University of Tokyo and Tokyo Metropolitan University. All participants were healthy, right-handed, had normal or corrected to normal vision, normal touch perception and no history of neurological or psychiatric conditions as assessed by self-report. All experiments involved different groups of participants. All participants had no preliminary knowledge about the RHI and the purpose of the experiment and gave written informed consent before the beginning of the experiments. The experiments were approved by the Ethics Committee in School of Engineering; The University of Tokyo followed the ethical standards laid down in the Declaration of Helsinki. Participants were reimbursed for their participation in the experiment with 1000 JPY per hour.

### Robotic master-slave system and virtual reality

**Figure 1:**
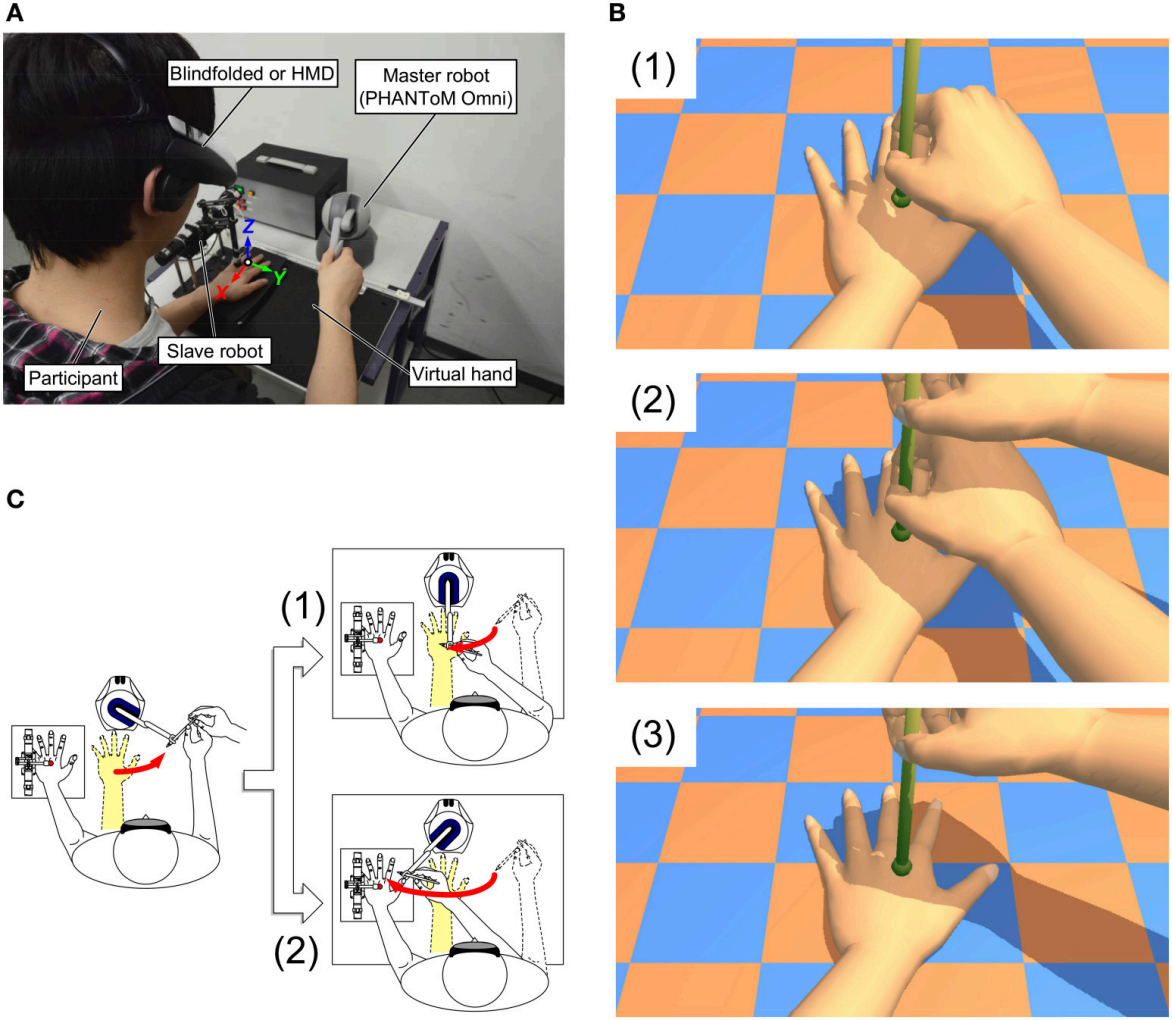
**Experimental setup and paradigm. (A)** The robotic master-slave systems as well as the experimental setup are shown. The 2-DOF slave robot is controlled by the master robot to apply touch (tapping) on the back of participants' left hands in the Z (vertical) direction (see Video 1). When the slave robot contacted the participants' left hands, the master robot (PHANToM Omni) produces a virtual force based on a defined virtual dynamics of the virtual hand (mass: *M* = 0.0 kg; viscosity: *D* = 0.0 Ns/mm; stiffness: *K* = 1.0 N/mm) via an impedance controller. **(B)** A virtual scenario rendered with OpenGL and GLEW was projected on the HMD in a stereoscopic view. (1) In the active self-touch conditions, the participants' left and right hands were presented through the HMD. (2) In the passive self-touch conditions, an experimenter's right hand was also presented above the participants' right hands as to mimic the action of the experimenter. (3) In the classical RHI stimulation (Experiment 3 only), the participants' right hands were removed from view (2). The movements of virtual right hands were linked to the master robot and the virtual left hand never moved. The distance between the physical left hand and the tapping hand (either one of the participants or the experimenter) was always 200 mm. **(C)** In Experiments 1 and 2, the drifts of the participants' right hands were measured. First, an experimenter moved the pen-type handle of master robot connected to the participants' right hands 350 mm away from their left hands. (1) Before stimulation, the participants were asked to return the handle to the position where their right hands were, whereas they pointed to the tapping position after stimulation. In addition to the measurement of right hand drift, the drift of left hand was measured in Experiment 3. (2) In the measurement of left hand drift, the participants were asked to indicate the felt position of their left middle fingers with the handle before and after each experimental block.

A robotic master-slave system (see Figure 1A) was used throughout all the experiments. The participants held the handle of a master robot with their right hands and manipulated it to interact with a virtual left hand (see Figure 1B). When the tip of the handle touched the virtual hand the master robot rendered a virtual stiffness at the participants' right hand based on the contact state. The movement of the master robot was sent to a slave robot, which applied a tactile stimulus to the participants' left hand in real time. Therefore, the participants could feel as if they were touching their own left hand.

A custom-made two-degrees-of-freedom (2-DOF) parallel-link robot was adopted as the slave robot. In the slave robot, two DC motors (RH-8D 6006, Harmonic Drive Systems), which had harmonic gear heads with 1/50 reduction ratio and optical encoders with 1000 ppr resolution, produced movement in the X and Z directions and sufficient contact force on the participants' hand. The DC motors were controlled by motor drivers (4-Q-DC Servoamplifier LSC 30/2, Maxon) based on command voltage from a multifunction data acquisition device (NI PCIe-6323, National Instruments) installed on a desktop computer. A plastic cap was attached to the tip of the slave robot so that the slave robot safely interacted with the participants' left hand. As for the master robot, we adopted a PHANToM Omni (Geomagic formally SensAble), which is a commercialized 3-DOF haptic device with a pen-type handle, because of its easy availability and good force-display function (maximum force: ~3.3 N); a previous experiment confirmed that the pen-type handle is intuitive for the participants to apply stroking and tapping to a rubber hand (Hara et al., 2011). The position sensing function of the master robot (resolution: ~0.055 mm) was employed to measure the proprioceptive drift (see Figure 1C). The master and slave robots were arranged in front of the participants so that the distance between the two robots was 200 mm (see Figure 1A). Instead of the rubber hand, a virtual hand was constructed with a force display function of the PHANToM Omni based on the position where the slave robot contacted the participants' left hand. To increase the self-touch experience, we used an impedance controller to simulate stiffness of the virtual hand (1.0 N/mm). The position of the slave robot was controlled on the basis of the movement of the master robot. The robot control was performed with 1 ms sampling time (i.e., 1 kHz sampling rate). Thus, the intrinsic delay of the robotic system was around 1 ms.

A head mounted display (HMD: HMZ-T1, Sony, resolution: 1280 × 720 pixels in each screen) was employed to display 3D graphics of virtual experimental environment (virtual hands and a virtual stick) in stereoscopic view in Experiments 2 and 3 (see Figure 1B); the 3D graphics were rendered by using OpenGL and GLEW. The estimated intrinsic delay of the HMD is ~33 ms. The behavior of the virtual right hand was rendered synchronously or asynchronously with the movement of the master robot manipulated by the participants (active stimulations) or an experimenter (passive or classical stimulations). During all three experiments, white noise was presented to the participants through headphones on the HMD to mask the noise generated by the master and slave robots.

### Dependent measures

#### Proprioceptive drift

We measured the effects of experimental manipulations on the change in proprioceptive sense of location (i.e., proprioceptive drift–PD) of participants' right, touch-administering (Experiments 1, 2, and 3) or left, touch-receiving hand (Experiment 3). The PD of the touch-administering hand was defined as a difference between the right hand location judgments before and after each experimental block. To obtain the pre-stimulation judgments, the experimenter lifted the participants' right hand, attached to the handle of the master robot, and positioned it ~150 mm rightwards from the initial position. The participants were then asked to point with their right hand (attached to the handle) to the location of the initial position. Similarly, to obtain the post-stimulation judgments, the experimenter displaced participants' right hand ~150 mm rightwards (or approximately 150 mm rightwards) from where they were tapping the virtual hand, and the participants were then asked to point to the location where they were touching the virtual hand during the experimental block (see Figure 1C). The pointed positions were measured by the position sensing function of the master device in a high precision. The PD for right hand was calculated as the difference between the pointed position before and after each experimental block.

When measuring the PD for the left, touch-receiving hand (Experiment 3), participants were asked before and after each experimental block to point with their right hand (attached to the handle of the master robot) to the felt position of their left middle finger (see Figure 1C). The PD was defined as the difference between the pre- and post-stimulation measures.

#### Illusion questionnaire

A 6-item questionnaire was used to measure the strength of experienced self-touch illusion. The items were adapted from the somatic-RHI questionnaire. The first two items referred to the sense of illusory self-touch (Q1–“I felt like I was tapping my left hand”) and the sense of agency (Q2–“The touch on my left hand matched the movements I made with my right hand”). The other items were unrelated to the bodily illusion and served as a control for suggestibility (Q3–“I felt like my left hand was becoming bigger”; Q4–“I couldn't feel my left hand”; Q5–“I felt as if I had more than one left hand”; Q6–“I felt my left hand was moving”). The participants were asked to designate on a 7-point Likert scale the strength of their agreement with each item (0 = “strongly disagree,” 6 = “strongly agree”). An item, referring to the sense of illusory ownership over the virtual hand (Q7–“I felt as if the virtual hand was my own left hand”), was added to the questionnaire in Experiments 2 and 3.

### Statistical analysis

The questionnaire ratings for each item as well as proprioceptive drift measures in each condition were averaged across trials for each participant. Due to deviation from normal distribution (Shapiro-Wilk test for normality), the averaged questionnaire ratings of each item from all three experiments were first analyzed with the Friedman test, and if significant, they were followed up with pairwise comparisons, using the 2-tailed Wilcoxon's signed rank test. Three planned comparisons were made for the data sets in Experiments 1 and 2, where the ratings of the synchronous conditions were compared with their respective asynchronous pair, and those of the two synchronous, but different mode of tactile stimuli administration, with each other. The level of significance was corrected for multiple comparisons using the Bonferroni method, where corrected α = 0.05∕3 = 0.0167. The planned comparisons of the questionnaire data in Experiment 3 were made by first comparing the ratings between synchronous and asynchronous conditions; and then, among active-synchronous, passive-synchronous, and classical-synchronous conditions. The α-level was therefore corrected for six comparisons using the Bonferroni method, resulting in corrected α = 0.05∕6 = 0.008. The PDs of each experiment were analyzed with repeated measures analysis of variance (ANOVA) with two within-participants factors: *Stimulation type* and *Synchrony*. When the sphericity was violated in Experiment 3 (Mauchly's test of sphericity), the repeated measures ANOVA *p*-values were corrected by Greenhouse-Geisser's epsilon (Bolton et al., 2007) and if significant, followed-up with pairwise comparisons, using 2-tailed, paired-sample *t*-tests. We used the same planned pairwise comparisons and the correction of the α-level as for the questionnaire data. One-sample, two-tailed *t*-test was used to verify whether the PDs significantly differed from zero (i.e., no drift).

## Experiments and results

### Experiment 1: Active and passive somatic RHI

#### Participants

Thirteen participants (three females) participated in Experiment 1. Their age ranged between 20 and 39 years (*M* = 27.0, *SD* = 6.5).

#### Experimental design and procedure

In Experiment 1 we investigated the effect of active (self-administered) stimulation on the sense of illusory self-touch using the somatic version of the RHI (somatic-RHI) paradigm (Ehrsson et al., 2005; White et al., 2010). In a 2 × 2 repeated measures design we manipulated *Stimulation type* (active vs. passive self-touch) and *Synchrony* (synchronous vs. asynchronous tactile stimulation).

Prior to the experiment (i.e., training session), participants were instructed how to manipulate the robotic device and were explained the general procedure of the experiment. During the experiment, blindfolded participants sat in front of a table with their left hand (palm down) placed on the base of the slave robot while holding the pen-type handle of the master robot with their right hand. In the active self-touch conditions, the participants manipulated the handle to tap a virtual left hand, created with a force display function at the level of the master robot, rendered 200 mm to the right from the participants' left hand. In the passive self-touch conditions, the experimenter guided the participants' right hand with the handle of the master robot to touch (tap) the virtual hand. In the synchronous conditions, the actuated movements and received tactile feedback were synchronous, whereas in the asynchronous conditions, a constant 500 ms delay was applied to the movement of the slave robot (Blanke et al., 2014), resulting in delayed tactile contact between the slave robot and participants' left hand. The 500 ms delay used in the asynchronous is an established method for the asynchronous condition in bodily illusions (e.g., Shimada et al., 2005; Tsakiris et al., 2007; Shimada et al., 2009; Blanke et al., 2014). In each experimental block, the tapping stimulation lasted 30 s. Each of the four conditions was repeated five times and presented to the participants in a randomized order. At the end of each experimental block, we first recorded the PD measurements and then administered the illusion questionnaire.

#### Illusion questionnaire ratings

The analysis of the questionnaire data using the Friedman test showed statistically significant differences between the conditions on the ratings of the illusory self-touch [Q1: “I felt like I was touching my left hand”; χ^2^(3) = 29.93, *p* < 0.001]. The *post-hoc* planned comparison showed that stronger self-touch illusion was experienced when the tactile stimulation was synchronous as compared to asynchronous in the active [synchronous: *M* = 4.37, *SD* = 1.48; asynchronous: *M* = 1.86, *SD* = 1.60; *Z* = −3.18, *p* = 0.001, α(corr.) = 0.0167, *r* = 0.62] as well as in the passive conditions [synchronous: *M* = 3.28, *SD* = 1.69; asynchronous: *M* = 1.58, *SD* = 1.64; *Z* = −3.11, *p* = 0.002, α(corr.) = 0.0167, *r* = 0.61]. Importantly, the experience of illusory self-touch was stronger in the active-synchronous than in the passive-synchronous conditions [*Z* = −2.80, *p* = 0.005, α(corr.) = 0.0167, *r* = 0.56].

Statistically significant differences between the conditions were also found for the ratings of the sense of agency [Q2: “The touch on my left hand matched the movements I made with my right hand”; χ^2^(3) = 33.52, *p* < 0.001]. The planned *post-hoc* comparisons revealed that the participants experienced stronger sense of agency when the tactile stimulation was synchronous in the active [synchronous: *M* = 5.26, *SD* = 0.84; asynchronous: *M* = 1.48, *SD* = 1.40; *Z* = −3.18, *p* = 0.001, α(corr.) = 0.0167, *r* = 0.62] as well as in the passive conditions [synchronous: *M* = 4.51, *SD* = 0.86; asynchronous: *M* = 1.12, *SD* = 1.19; *Z* = −3.18, *p* = 0.001, α(corr.) = 0.0167, *r* = 0.62]. As predicted, during the synchronous stimulation, the sense of agency was enhanced in the active as compared to the passive condition [*Z* = −2.84, *p* = 0.005, α(corr.) = 0.0167, *r* = 0.56].

The ratings of the other four items were low (*M* < 0.50, *SD* < 0.70) and did not significantly differ across the four conditions [Q3: χ^2^(3) = 0.82, *p* = 0.845; Q4: χ^2^(3) = 1.90, *p* = 0.593; Q5: χ^2^(3) = 4.16, *p* = 0.245; Q6: χ^2^(3) = 0.97, *p* = 0.809].

#### Proprioceptive drift of the touch-administering hand

Statistical analyses for proprioceptive drift of the touch-administering hand showed no significant main effects of *Stimulation type* [active: *M* = 2.34, *SD* = 5.65; passive: *M* = 2.21, *SD* = 8.25; *F*_(1, 12)_ = 0.00, *p* = 0.956, η^2^ = 0.00] and *Synchrony* [synchronous: *M* = 3.21, *SD* = 6.84; asynchronous: *M* = 1.34; *SD* = 5.16; *F*_(1, 12)_ = 2.69, *p* = 0.127, η^2^ = 0.18]. Statistically insignificant was also the interaction between the two factors [*F*_(1, 12)_ = 0.06, *p* = 0.813, η^2^ = 0.01]. Additionally, one-sample two-tailed *t*-tests showed that the mean PDs did not significantly differ from zero (i.e., no drift) in any of the experimental conditions [active-synchronous: *M* = 3.47, *SD* = 8.11; *t*_(12)_ = 1.54, *p* = 0.149; active-asynchronous: *M* = 1.21, *SD* = 5.75, *t*_(12)_ = 0.76, *p* = 0.464; passive-synchronous: *M* = 2.95, *SD* = 10.10, *t*_(12)_ = 1.05, *p* = 0.313; passive-asynchronous: *M* = 1.47, *SD* = 7.13, *t*_(12)_ = 0.75, *p* = 0.470].

**Figure 2:**
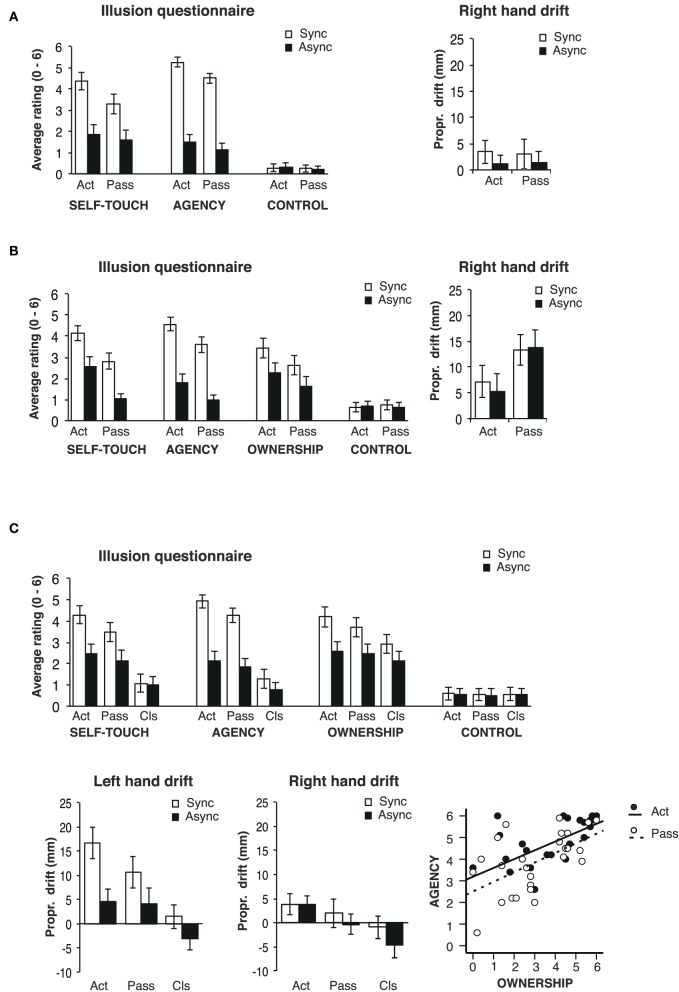
**Mean questionnaire ratings and proprioceptive drifts. (A)** Mean questionnaire ratings and mean PDs for right hand in Experiment 1. The sense of illusory self-touch was enhanced in synchronous stimulation compared to asynchronous stimulation. Additionally, the participants reported stronger illusion when they actively touched the virtual hand and their own hands with the master-slave system. Neither synchrony nor stimulation type modulated the PD. **(B)** Mean questionnaire ratings and mean PDs for right hand in Experiment 2. In addition to the sense of illusory self-touch, the participants reported stronger illusory ownership over the virtual left hand with synchronous and active stimulation. Greater PD was found in the passive stimulation conditions. **(C)** Mean questionnaire ratings, PDs for both left and right hands in Experiment 3, and correlation between the sense of agency and sense of hand ownership for active-synchronous and passive-synchronous conditions. The illusory self-touch was induced in active and passive self-touch, and all the stimulation types allowed the participants to experience the illusory ownership over the virtual left hand. The experience of RHI became stronger in the order of active self-touch, passive self-touch, and classical stimulation. The PDs for right hand did not show any significance, but a greater PD for left hand was observed with synchronous stimulation in active self-touch. In the graphs, Act/Pass/Cls and Sync/Async mean active self-touch/passive self-touch/classical stimulation and synchronous/asynchronous, respectively.

Thus, the results of Experiment 1 (Figure 2A) indicated that synchronous active self-touch elicited stronger illusory self-touch than synchronous passive self-touch in the absence of visual feedback.

### Experiment 2: Active and passive visual RHI

#### Participants

Fifteen participants (10 females) took part in Experiment 2. Their age ranged between 18 and 41 years (*M* = 23.9, *SD* = 5.6).

#### Experimental design and procedure

In Experiment 2, we investigated the effect of active (self-administered) stimulation on the strength of self-touch illusion and sense of illusory ownership over the virtual hand. Thus, we adapted and combined the somatic-RHI and classical RHI paradigms for the use in the virtual reality and robotic setting. As in Experiment 1, we manipulated *Stimulation type* and *Synchrony* in a 2 × 2 repeated measures design. We readapted the experimental procedure from Experiment 1 by adding visual cues of the virtual hands. Thus, a virtual scenario matching the experimental manipulation was projected through the HMD [see Figure 1B (1) for active conditions and Figure 1B (2) for passive conditions]. Similar to Experiment 1, each experimental block lasted 30 s, the order of the four conditions was randomized across the participants and each condition was repeated five times. The PD for the right, touch-administering hand was measured in the same manner as in Experiment 1 and the initial position of the participants' right hand matched the position of the virtual left hand projected onto the HMD. During the measurement of right hand localization, the virtual hands were not displayed on the HMD. At the end of each experimental block the participants also answered to the illusion questionnaire used in Experiment 1, which now also included an additional item referring to the sense of illusory ownership for the virtual hand (Q7–“I felt as if virtual hand was my own left hand”).

#### Illusion questionnaire ratings

The ratings of the illusory self-touch significantly differed between the four conditions [Q1: “I felt like I was touching my left hand”; χ^2^(3) = 31.71, *p* < 0.001]. The planned pairwise comparisons showed that synchronous tactile stimulation increased the self-touch illusion in the active [synchronous: *M* = 4.15, *SD* = 1.45; asynchronous: *M* = 2.56, *SD* = 1.78; *Z* = −2.75, *p* = 0.006, α(corr.) = 0.0167, *r* = 0.50] as well as in the passive conditions [synchronous: *M* = 2.82, *SD* = 1.52; asynchronous: *M* = 1.05, *SD* = 0.97; *Z* = −3.41, *p* = 0.001, α(corr.) = 0.0167, *r* = 0.62]. During the synchronous stimulation, the participants reported stronger self-touch illusion when the tactile stimuli were actively applied to the hands [*Z* = −3.33, *p* = 0.001, α(corr.) = 0.0167, *r* = 0.61].

The ratings of the sense of agency also significantly differed across the four conditions [Q2: “The touch on my left hand matched the movements I made with my right hand”; χ^2^(3) = 33.41, *p* < 0.001]. The follow-up planned pairwise comparisons showed that the sense of agency was rated stronger after the synchronous than the asynchronous tactile stimulation in the active [synchronous: *M* = 4.56, *SD* = 1.26; asynchronous: *M* = 1.83, *SD* = 1.52; *Z* = −3.10, *p* = 0.002, α(corr.) = 0.0167, *r* = 0.57] as well as in the passive conditions [synchronous: *M* = 3.61, *SD* = 1.46; asynchronous: *M* = 0.97, *SD* = 0.83; *Z* = −3.41, *p* = 0.001, α(corr.) = 0.0167, *r* = 0.62]. As we predicted, active tactile administration in the synchronous condition resulted in stronger sense of agency than in the passive-synchronous conditions [*Z* = −3.19, *p* = 0.001, α(corr.) = 0.0167, *r* = 0.58].

The Friedman test showed statistically significant differences in the ratings of illusory ownership over the virtual hand across the four conditions [Q7: “I felt as if virtual hand was my own left hand”; χ^2^(3) = 20.90, *p* < 0.001]. The planned *post-hoc* comparisons showed that the sense of illusory ownership was stronger when the tactile stimulation was synchronous in both, active [synchronous: *M* = 3.43, *SD* = 1.77; asynchronous: *M* = 2.28, *SD* = 1.82; *Z* = −2.44, *p* = 0.015, α(corr.) = 0.0167, *r* = 0.44] and passive conditions [synchronous: *M* = 2.62, *SD* = 1.73; asynchronous: *M* = 1.65, *SD* = 1.64; *Z* = −2.79, *p* = 0.005, α(corr.) = 0.0167, *r* = 0.50]. Importantly, the active stimulation increased the illusory ownership of the virtual hand as compared to the passive stimulation in the synchronous conditions [*Z* = −2.45, *p* = 0.014, α(corr.) = 0.0167, *r* = 0.45].

The ratings of the other four items were low (*M* = 1.80, *SD* < 1.80) and were not significantly modulated by the experimental conditions [Q3: χ^2^(3) = 2.46, *p* = 0.482; Q4: χ^2^(3) = 0.80, *p* = 0.849; Q5: χ^2^(3) = 2.17, *p* = 0.538; Q6: χ^2^(3) = 0.19, *p* = 0.980].

#### Proprioceptive drift of the touch-administering hand

Statistical analyses showed a significant main effect of *Stimulation type* [active: *M* = 6.26, *SD* = 10.49; passive: *M* = 13.52, *SD* = 11.65; *F*_(1, 14)_ = 15.28, *p* = 0.002, η^2^ = 0.52] but no significant main effect of *Synchrony* [synchronous: *M* = 10.25, *SD* = 9.58; asynchronous: *M* = 9.52, *SD* = 12.61; *F*_(1, 14)_ = 0.13, *p* = 0.726, η^2^ = 0.01] and no significant interaction between these two factors [*F*_(1, 14)_ = 0.26, *p* = 0.617, η^2^ = 0.02]. One-sample two-tailed *t*-tests revealed that the mean PDs significantly differed from zero in all experimental conditions [active-synchronous: *M* = 7.19, *SD* = 11.89; *t*_(14)_ = 2.34, *p* = 0.035; passive-synchronous: *M* = 13.31, *SD* = 11.41, *t*_(14)_ = 4.52, *p* < 0.001; passive-asynchronous: *M* = 13.72, *SD* = 13.16, *t*_(14)_ = 4.04, *p* = 0.001], except in one experimental condition [active-asynchronous: *M* = 5.33, *SD* = 13.51, *t*_(14)_ = 1.53, *p* = 0.149].

The results of Experiment 2 (Figure 2B) indicated that with visual feedback, synchronous active self-touch elicited stronger illusory self-touch and sense of ownership over the virtual hand than synchronous passive self-touch. No synchrony-related PD was found for the touch-administering hand.

### Experiment 3: Comparison of active, passive, and classical RHI

#### Participants

Twelve participants (six females) participated in Experiment 3. Their age ranged between 21 and 32 years (*M* = 25.8, *SD* = 2.6).

#### Experimental design and procedure

In Experiment 3, we directly compared the role of experimental factors investigated in Experiments 1 and 2 to the classical RHI condition (touch completely administrated by an experimenter). Thus, we used a 3 × 2 factorial design with the within-participants factors of *Stimulation type* (active self-stimulation, passive self-stimulation, and classical tactile stimulation) and *Synchrony* (synchronous vs. asynchronous). Importantly, in this last experiment, we measured the PDs for both right and left hands. During each experimental block, participants viewed an appropriate virtual scenario through the HMD (Figure 1B). Similar to Experiments 1 and 2, each experimental block lasted 30 s, and six conditions were randomly presented and repeated 10 times; this yielded a total of 60 experimental blocks. At the end of each experimental block, the participants responded to the same questionnaire used in Experiment 2. The measurement of right hand localization was performed as in Experiments 1 and 2, with the only difference that we asked the participants to localize the position in which they saw the experimenter's tapping in the classical RHI experimental blocks (as the participants never moved their hands). In Experiment 3, we additionally measured the drifts of participants' left hand. Thus, before and after the experimental manipulation, the participants were asked to move their right hand (attached to the handle of the master robot) so as to point to the felt position of their left middle fingers (see Figure 1C). Both the right and left drifts were measured in two measurement orders (i.e., first for right/left hand drifts and second for left/right hand drifts) in each experimental block. Each measurement order was presented five times and was balanced across participants.

#### Illusion questionnaire ratings

We first investigated the effects of active and passive self-touch as well as the classical RHI (tactile stimulation administered by an experimenter) on subjective ratings (see Figure 2C). The Friedman test showed that the ratings of the illusory self-touch significantly differed between the experimental conditions [Q1: “I felt like I was touching my left hand” χ^2^(5) = 50.09, *p* < 0.001]. The planned pairwise comparisons revealed that the illusory self-touch was experienced significantly stronger in the active-synchronous (*M* = 4.29, *SD* = 1.51) than in the active-asynchronous conditions [*M* = 2.47, *SD* = 1.55; *Z* = −3.06, *p* = 0.002, α(corr.) = 0.008, *r* = 0.63]. The ratings of illusory self-touch were also significantly higher in the passive-synchronous (*M* = 3.48, *SD* = 1.54) as compared to the passive-asynchronous condition [*M* = 2.15, *SD* = 1.72; *Z* = −2.65, *p* = 0.008, α(corr.) = 0.008, *r* = 0.53]. As predicted, low scores and no difference in the self-touch ratings between synchronous (*M* = 1.08, *SD* = 1.43) and asynchronous stimulation (*M* = 0.98, *SD* = 1.45) were found in the classical RHI tactile stimulation [*Z* = −0.42, *p* = 0.673, α(corr.) = 0.008, *r* = 0.09]. Moreover, the ratings of the illusory self-touch in the active-synchronous condition were significantly higher than the ratings in the passive-synchronous [*Z* = −2.937, *p* = 0.003, α(corr.) = 0.008, *r* = 0.60] and classical-synchronous conditions [*Z* = −3.062, *p* = 0.002, α(corr.) = 0.008, *r* = 0.63], and ratings in the passive-synchronous condition were significantly higher than the ratings in the classical-synchronous condition [*Z* = −3.06, *p* = 0.002, α(corr.) = 0.008, *r* = 0.62].

Significant differences between the experimental conditions were also found for the ratings of the sense of agency [Q2: “The touch on my left hand matched the movements I made with my right hand”; χ^2^(5) = 46.68, *p* < 0.001]. The planned pairwise comparisons revealed that the sense of agency was experienced significantly stronger in the active-synchronous (*M* = 4.92, *SD* = 1.07) than in the active-asynchronous conditions [*M* = 2.12, *SD* = 1.69; *Z* = −3.06, *p* = 0.002, α(corr.) = 0.008, *r* = 0.62]. Similarly, the passive-synchronous stimulation (*M* = 4.24, *SD* = 1.18) resulted in higher ratings than the passive-asynchronous stimulation [*M* = 1.83, *SD* = 1.47; *Z* = −3.06, *p* = 0.002, α(corr.) = 0.008, *r* = 0.62], whereas the synchrony of tapping did not affect the sense of agency in the classical RHI condition (synchronous: *M* = 1.28, *SD* = 1.53; asynchronous: *M* = 0.75, *SD* = 1.29; *Z* = −1.95, *p* = 0.051, α(corr.) = 0.008, *r* = 0.40]. Moreover, the sense of agency was stronger in the active-synchronous than in the passive-synchronous (*Z* = −2.65, *p* = 0.008, α(corr.) = 0.008, *r* = 0.54] and classical-synchronous conditions [*Z* = −3.06, *p* = 0.002, α(corr.) = 0.008, *r* = 0.62]. The sense of agency was also rated stronger in the passive-synchronous as compared to the classical-synchronous condition [*Z* = −3.06, *p* = 0.002, α(corr.) = 0.008, *r* = 0.63].

The Friedman test also detected statistically significant differences between the experimental manipulations in the ratings of illusory ownership [Q7: “I felt as if the virtual hand was my own left hand”; χ^2^(5) = 44.56, *p* < 0.001]. The planned *post-hoc* comparisons showed that the synchrony of tapping significantly increased the ratings of illusory ownership when the type of stimulation was active [active-synchronous: *M* = 4.18, *SD* = 1.63; active-asynchronous: *M* = 2.58, *SD* = 1.61; *Z* = −3.06, *p* = 0.002, α(corr.) = 0.008, *r* = 0.63] as well as passive self-touch [passive-synchronous: *M* = 3.72, *SD* = 1.60; passive-asynchronous: *M* = 2.44, *SD* = 1.66; *Z* = −2.98, *p* = 0.003, α(corr.) = 0.008, *r* = 0.61] but not in the classical RHI tactile stimulation [classical-synchronous: *M* = 2.93, *SD* = 1.52; classical-asynchronous: *M* = 2.12, *SD* = 1.67; *Z* = −2.19, *p* = 0.028, α(corr.) = 0.008, *r* = 0.45]. When the stimulation was synchronous active self-touch resulted in higher ownership ratings than passive self-touch [*Z* = −3.066, *p* = 0.002, α(corr.) = 0.008, *r* = 0.63] or touch administered by the experimenter in the classical RHI [*Z* = −3.06, *p* = 0.002, α(corr.) = 0.008, *r* = 0.63]. The hand ownership ratings were also higher in the passive-synchronous as compared to classical-synchronous condition [*Z* = −2.91, *p* = 0.004, α(corr.) = 0.008, *r* = 0.59].

No significant differences between the experimental conditions were found for the ratings of the other four questionnaire items [Q3: χ^2^(5) = 5.37, *p* = 0.373; Q4: χ^2^(5) = 7.52, *p* = 0.185; Q5: χ^2^(5) = 9.88, *p* = 0.079; Q6: χ^2^(5) = 8.04, *p* = 0.154].

#### Proprioceptive drift of the touch-administering hand

The ANOVA showed that neither *Stimulation type* [active: *M* = 4.54, *SD* = 7.89; passive: *M* = 3.28, *SD* = 4.53; classical: *M* = 1.55, SD = 7.80; *F*_(1.26, 13.88)_ = 0.56, *p* = 0.581, η^2^ = 0.05] nor *Synchrony* [synchronous: *M* = 2.81, *SD* = 5.50, asynchronous: *M* = 3.43, *SD* = 4.35; *F*_(1, 11)_ = 0.13; *p* = 0.730; η^2^ = 0.01] significantly affected the PD of the participants' right (i.e., touch-administering) hand. Interaction between *Stimulation type* and *Synchrony* was also not significant [*F*_(2, 22)_ = 1.84; *p* = 0.183; η^2^ = 0.14].

#### Proprioceptive drift of the touch-receiving hand

The analysis of the PD of the left, touch-receiving hand showed a statistically significant main effect of *Stimulation type* [active: *M* = 10.57, *SD* = 8.66, passive: *M* = 7.40, *SD* = 7.84, classical: *M* = −0.77, *SD* = 5.97; *F*_(2, 22)_ = 6.60, *p* = 0.006; χ^2^ = 0.38] and *Synchrony* [synchronous: *M* = 9.54, *SD* = 6.52, asynchronous: *M* = 1.93, *SD* = 5.75; *F*_(1, 11)_ = 7.89, *p* = 0.017; χ^2^ = 0.42] on the PD of the participants' left (i.e., touch-receiving) hand. The interaction between the two experimental manipulations was not statistically significant [*F*_(2, 22)_ = 1.59; *p* = 0.23; χ^2^ = 0.13]. A *post-hoc* analysis of planned comparisons using a paired-sample two-tailed *t*-test showed that the PD after active and synchronous self-touch (active-synchronous: *M* = 16.56, *SD* = 11.27) was significantly larger than the PD after the active and asynchronous self-touch [active-asynchronous: *M* = 4.57, *SD* = 8.58, *t*_(11)_ = 4.13, *p* = 0.002, α(corr.) = 0.008, *r* = 0.78] and larger than the PD after the synchronous tactile stimulation in the classical RHI [classical-synchronous: *M* = 1.45, *SD* = 8.47; *t*_(11)_ = 4.48, *p* = 0.001, α(corr.) = 0.008, *r* = 0.80]. The other planned comparisons did not yield statistically significant differences (all *p* > 0.008).

#### Correlation between the sense of agency and hand ownership

In order to investigate the strength of the relationship between the sense of agency and hand ownership, we combined and correlated the ratings of the sense of agency (Q2) and illusory hand ownership (Q7) in the synchronous tactile stimulation from Experiments 2 and 3. The analysis revealed a strong positive relationship when the type of the tactile stimulation was active [Pearson's *r*_(27)_ = 0.597, *p* = 0.001, α(corr.) = 0.025] as well as when it was passive [Pearson's *r*_(27)_ = 0.569, *p* = 0.002, α(corr.) = 0.025].

## Discussion

### Illusory self-touch and illusory ownership

The present experiments investigated the induction of the well-established RHI, using active self-touch. We used a novel adaptation of the RHI employing a robotic master-slave system, which allowed participants to induce the tactile stimulation actively, thus introducing movement related efferent information to the illusion. Collectively, the results showed three main findings: First, active self-touch compared to passive self-touch increased subjective scores of illusory self-touch (Experiments 1 and 2) and also of illusory ownership (Experiment 2) in the somatic and visual versions of the RHI. Second, both active and passive self-touch increased illusory ownership of the virtual hand compared to the classical tactile only induction of the RHI (Experiment 3). Finally, proprioceptive drift, an objective measure of illusory body ownership was found only for the touch-receiving hand (Experiment 3) and was larger for active self-touch compared to the classical tactile RHI condition.

### Agency and illusory ownership

While body ownership has classically been related to multisensory integration of passive sensory signals (Botvinick and Cohen, 1998; Ehrsson et al., 2005; Tsakiris and Haggard, 2005; Blanke, 2012) recent research has shown that sensorimotor correlations based on efferent signals provide important information for body ownership (Dummer et al., 2009; Kammers et al., 2009; Kalckert and Ehrsson, 2012; Suzuki et al., 2013), suggesting a role of motor signals in the formation of body representations. Experiments using matching visuo-motor stimulations have shown that these induce sensations of body ownership for limbs as well as bodies (Sanchez-Vives et al., 2010; Walsh et al., 2011; Banakou et al., 2013; Rognini et al., 2013). Here we go beyond visuo-motor correlations to show that motor signals present in active self-touch increase illusory ownership of a hand with respect to passive self-touch (proprioceptive signals) and classical RHI (tactile only). This finding expands previous works showing that passive multisensory visuo-tactile integration induces illusory ownership (Botvinick and Cohen, 1998; Tsakiris and Haggard, 2005; Costantini and Haggard, 2007; Ionta et al., 2011; Salomon et al., 2013b) by showing that efferent motor information is integrated and enhances the illusion. From a theoretical perspective, this is in line with accounts suggesting that action and motor signals have an important role in the formation of the sense of bodily self (Knoblich, 2002; van den Bos and Jeannerod, 2002; Blakemore and Frith, 2003; Schütz-Bosbach et al., 2006; Tsakiris et al., 2006; Salomon et al., 2009, 2013a). Furthermore, these findings show that the efferent motor information is integrated with not only visual signals as in previous visuo-motor RHI studies (Dummer et al., 2009; Kammers et al., 2009; Sanchez-Vives et al., 2010; Kalckert and Ehrsson, 2012, 2014; Banakou et al., 2013; Banakou and Slater, 2014) but also afferent proprioceptive and tactile signals which are thought to be central to body ownership (Blanke, 2012; Palluel et al., 2012). We suggest that this increase in the subjective aspect of the illusion is due to the additional information provided by the efferent motor signals of self-authored movements. These signals bolstering correspondence between the afferent sensory inputs and the predicted sensory consequences of the self-generated action provide an important source of self-related information (Tsakiris et al., 2005, 2006; Rognini et al., 2013; Salomon et al., 2013a) which in turn affects the incorporation of the virtual hand to the self. Interestingly, previous studies of self-touch have shown that tactile signals associated with self-touch are suppressed both at the behavioral level (Blakemore et al., 2000) and in the brain (Blakemore et al., 1998; Bays et al., 2006; Dieguez et al., 2009; Shergill et al., 2012; Martuzzi et al., 2015). While this suppression of tactile perception may seem to suggest that self-touch should have lower impact on a tactile based bodily illusion, we advocate an opposing interpretation, namely that the same predictive models underlying the tactile attenuation are also responsible for the increase in illusory ownership. Recent theories have suggested an important role for predictive coding (Friston, 2010) in establishing a model of the self (Clark, 2013; Apps and Tsakiris, 2014). These theories propose that the self is constructed through a Bayesian computation minimizing the prediction errors (incongruences between predicted and incoming sensory signals). Here, the addition of efferent information through active self-touch introduces further predictive signals that, when matched with the tactile sensations, strengthen the illusion that the hand belongs to the self. The efferent information, which is related to self-authored movements, may allow a reduction of the prediction errors though the increased sensorimotor correspondences. Thus, the convergence of multiple signals may enhance the illusory ownership. This is supported by our data, which shows a hierarchy of illusory embodiment based on the availability of motor (active), proprioceptive (passive) or tactile only (classical RHI) conditions.

### Mislocalization of the hand

The RHI is typically associated with a mislocalization of the stimulated hand toward the fake hand, often termed “proprioceptive drift” (Botvinick and Cohen, 1998; Costantini and Haggard, 2007; but see Rohde et al., 2011; Davies et al., 2013). Our novel robotic setup allowed precise and well controlled measurements of proprioceptive drift. Recently it has been proposed that such proprioceptive drift may be also presented on the hand administering the tactile stroking in the somatic-RHI (White et al., 2011). Our results (Experiment 1) did not replicate these findings of drift on the touch-administering hand during the somatic-RHI. However, the two studies differed in several aspects relating to the measurement of the proprioceptive drift. It is possible that differences in the experimental setup (virtual hand here vs. rubber hand in White et al., 2011), duration of stimulation (30 s vs. 180 s) or the mode of measurement (pointing vs. visual perceptual judgments) may account for this difference. In Experiments 2 and 3 using a visual version of the RHI no synchrony-modulated proprioceptive drift was found for the touch-administering hand. In contrast, the results of Experiment 3 in which proprioceptive drift was measured for both the administering and stimulated hand show a synchrony-modulated difference for the active condition for the tapped hand. Critically, the drift of the touched hand in the active self-touch condition was larger than that elicited by the classical tactile RHI, mirroring the effects found in the subjective feeling of illusory ownership. The results of the proprioceptive drift, an implicit measure of body ownership, show that active self-touch induces a larger mislocalization of the hand compared to tactile stimulation alone, suggesting that such proprioceptive error may also be affected by efferent predictive signals. A similar result has also been recently found using active self-touch in the context of the Full Body Illusion where synchronous active touch caused changes in subjective experience (Hara et al., 2014) and a larger proprioceptive drift of the full body compared to asynchronous tactile feedback (Blanke et al., 2014).

## Conclusion

In a series of three studies using a novel robotic setup we have shown that active self-touch induces higher illusory ownership over a virtual hand as measured by subjective explicit, as well as objective implicit measures. Higher illusory self-touch was induced for both somatic and visual variants of the RHI, indicating that it is not dependent on visual feedback. Extending the results of previous studies on active movements in shaping our sense of bodily self (Tsakiris et al., 2006; Dummer et al., 2009; Kammers et al., 2009; Kalckert and Ehrsson, 2012; Suzuki et al., 2013), our results highlight the role of the correspondence between efferent motor signals and afferent sensory inputs in building our sense of body ownership. Thus, self-touch may have a special role in the formation of our bodily self-representation.

### Conflict of interest statement

The authors declare that the research was conducted in the absence of any commercial or financial relationships that could be construed as a potential conflict of interest.

## Supplementary material

Video 1 **Demo movie about active self-touch: 162293_Hara_Video1.WMV**.
