# Erratum: Voluntary Self-touch Increases Body Ownership

**DOI:** 10.3389/fpsyg.2015.01786

**Published:** 2015-11-17

**Authors:** Frontiers Production Office

**Affiliations:** Frontiers Production Office, FrontiersLausanne, Switzerland

**Keywords:** sense of body ownership, sense of agency, self-touch, rubber hand illusion, multisensory integration, volition, robotics and haptic technology

Reason for Erratum:

Due to a typesetting error, elements of Figure 1, panel C were partially cropped. The publisher apologizes for this error and the correct version of Figure 1 appears below.

**Figure 1:**
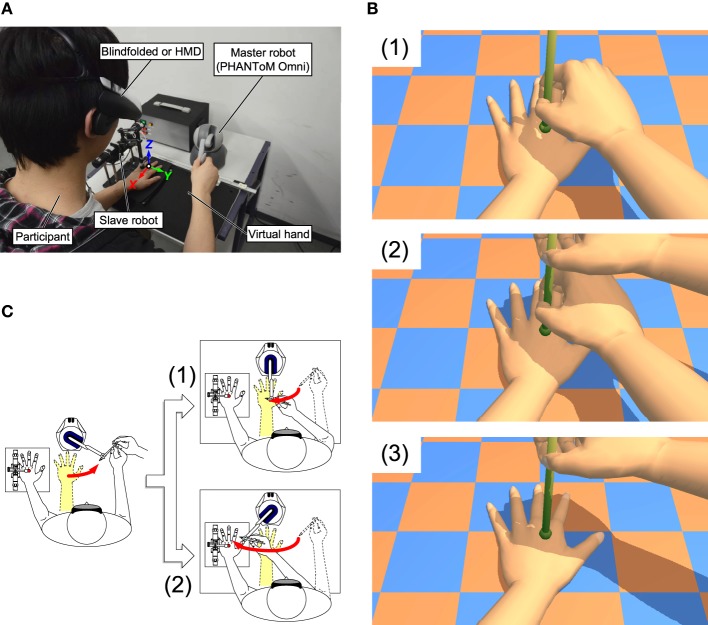
**Experimental setup and paradigm**.

This error does not change the scientific conclusions of the article in any way.

Original article has been updated.

